# Resveratrol promotes osteoblastic differentiation in a rat model of postmenopausal osteoporosis by regulating autophagy

**DOI:** 10.1186/s12986-020-00449-9

**Published:** 2020-04-16

**Authors:** Wei Wang, Li-Mei Zhang, Chang Guo, Jian-Feng Han

**Affiliations:** 1 Department of Endocrinology, the Second Affiliated Hospital of Harbin Medical University, Harbin, 150086 China; 2 Endocrinology Laboratory, the Second Affiliated Hospital of Harbin Medical University, Harbin, 150086 China; 3 Department of Orthopaedics, the Second Affiliated Hospital of Harbin Medical University, No. 246 Xuefu Road, Harbin, 150086 Heilongjiang China

**Keywords:** Resveratrol, Osteoblasts, Osteoclasts, Autophagy

## Abstract

**Objective:**

Resveratrol is a natural polyphenolic compound that ameliorates postmenopausal osteoporosis by activating the estrogen receptor. Research has shown that resveratrol exhibits some type of estrogen receptor agonist activity, reducing the risk of breast cancer. However, its mechanism of action remains largely unknown. This study aims to investigate the effect of resveratrol on osteoblastic and osteoclastic differentiation and its potential role in the regulation of autophagy.

**Methods:**

Sprague Dawley (SD) rats underwent ovariectomies (OVX) and were administered resveratrol (at 10, 20 or 40 mg/kg/d) for 8 weeks. The calcium content and the bone mineral density (BMD) were measured in the lumbar vertebrae (L3) and the right distal femur-tibia bone region. The osteoblasts and osteoclasts were isolated from rat lumbar vertebrae by enzyme digestion and bone marrow induction, respectively. The cells were then cultured with resveratrol in combination with bafilomycin or leupeptin to inhibit or activate autophagy, respectively. Western blotting was used to assess the differentiation markers and autophagy-related genes in the osteoblasts and osteoclasts.

**Results:**

Compared to the sham group, the bone calcium content and BMD were significantly decreased in the OVX group (*p* < 0.05), while resveratrol attenuated these in a dose-dependent manner. In the osteoblasts, vascular endothelial growth factor (VEGF), and alpha-1 type I collagen (COL1A1) were markedly decreased, and in osteoclasts, the receptor activator of nuclear factor-κB ligand (RANKL) was increased in the OVX group, while resveratrol reversed this pattern in a dose-dependent manner. The inhibition of autophagy in osteoblasts and its activation in osteoclasts was observed in the OVX group. However, with resveratrol, this was reversed in a dose-dependent manner.

**Conclusion:**

Overall, resveratrol promotes osteoblastic differentiation and suppresses osteoclastic differentiation in a rat model with postmenopausal osteoporosis by regulating autophagy.

## Background

Postmenopausal osteoporosis is a metabolic disease in which the endocrine function of the ovary becomes imbalanced and declines, leading to significantly decreased estrogen levels, which results in more active bone resorption from the osteoclasts than bone formation from the osteoblasts [1]. Postmenopausal osteoporosis is characterized by a progressive systemic bone mineral density (BMD) reduction and bone microstructure changes [2]. Osteoporosis is the most common cause of fractures in elderly women. The most vulnerable sites affected are the spine, hip and distal radius [3]. The incidence of postmenopausal osteoporosis in women in developed countries is approximately 38%, and it usually does not have a specific clinical manifestation until the patient suffers a stress-induced fracture [4]. The causes of postmenopausal osteoporosis are multifactorial. Existing studies suggest that an imbalance between bone formation and resorption caused by estrogen deficiency is the most crucial factor. With estrogen deficiency, the increase in bone resorption is greater than that of bone formation resulting in net bone loss [5–7]. Osteoblasts communicate with osteoclasts via direct contact. When the two cells are in contact, they form intercellular connections called gap junctions, allowing small water-soluble molecules to pass between the two cell types [8]. Osteoblasts are known to secrete macrophage colony-stimulating factors (M-CSF), monocyte chemoattractant protein-1 (MCP-1), and function through the osteoprotegerin (OPG)/receptor activator of nuclear factor-κB ligand (RANKL)/RANK, LGR4/RANKL/RANK, Ephrin2/ephB4, and Fas/FasL pathways in order to form new bone [9]. RANKL is the only factor known to induce the differentiation, development, and function of osteoclasts. By binding to RANK, RANKL not only promotes osteoclast differentiation, but also activates mature osteoclasts in a dose-dependent manner, increasing the bone resorption capacity [10, 11]. OPG, a decoy receptor for RANKL, can bind to RANKL ligand in osteoblast/stromal cells, thus blocking the RANKL-RANK ligand interaction between osteoblast/stromal cells and osteoclast precursors, which suppresses the differentiation of the osteoclast precursor into a mature osteoclast, and inhibits bone resorption [12]. On the other hand, osteoclasts regulate bone formation via the d2 isoform of vacuolar (H+) ATPase (v-ATPase) V0 domain (Atp6v0d2), complement component 3a, semaphorin 4D or microRNAs [9]. Hence, investigating the mechanisms of postmenopausal osteoporosis and its related signaling pathways is important for the development of clinical treatments.

Autophagy is an important physiological process for maintaining cell homeostasis by eliminating damaged organelles and proteins. There are three types of autophagy, namely macroautophagy, microautophagy and chaperone⁃mediated autophagy. Macroautophagy is the most common type of autophagy [13]. Recently, increasing evidence suggests that autophagy plays an important role in maintaining the balance of bone metabolism. For this reason, the modulation of autophagy is a crucial factor for osteoporosis. However, the regulation of the mechanism of autophagy in osteoporosis is still unclear [14].

There are approximately 30 autophagy related-proteins (Atgs) [15]. Importantly, Atg7 is necessary for the Atg12 to bind to Atg5, which acts as an E1-like enzyme. The conjugation of phosphatidylethanolamine (PE) to LC3 is mediated by Atg3 and the Atg5–Atg12 complex, which functions as an E2-like and E3-like enzyme, respectively, which is coupled with the translocation of LC3 from the cytosol to the isolation membrane. Thus, this translocation is deemed to be a reliable marker of autophagy [15]. A recent in vivo study revealed that the selective knockout of Atg7 in monocytes markedly inhibited the formation of osteoclasts and bone loss by suppressing autophagy [16]. In addition, high doses of glucocorticoid reduce the number of LC3B positive osteoblasts, thereby attenuating osteogenic activity [17]. To further validate the role of autophagy in osteoporosis, the key gene for autophagosome formation in osteoblasts was knocked out, and this led to osteopenia or osteoporosis in rats. Additionally, the osteoporosis was significantly reversed when the rats were fed the autophagy agonist rapamycin [18]. In a human study, the gene chip investigation from 984 volunteers showed that the autophagy-related gene (ATG) was the only statistically significant indicator associated with BMD in the distal radius [19]. These results all suggested that the autophagy signaling pathway played an important function in the pathogenesis of osteoporosis.

Estrogen replacement therapy is reported to have beneficial effects on postmenopausal osteoporosis. However, certain medications used for replacement therapy have several serious side effects, such as an increased risk of breast cancer and uterine cancer [20]. Therefore, finding new therapeutic strategies for postmenopausal osteoporosis, with fewer side effects, is necessary. Resveratrol is a natural polyphenolic compound that has estrogen activity. It is reported to function as an estrogen replacement therapy option and has beneficial bone protection. Moreover, it reduces the risk of breast cancer [21]. The underlying mechanism of resveratrol for postmenopausal osteoporosis treatment is not completely understood. It is reported that resveratrol increases the expression of Runx2, decreases the expression of PPAR-γ and inhibits PPAR-γ activity by mediating NCoR, resulting in the promotion of the osteogenic differentiation of mesenchymal stem cells [22]. In the present study, we explored the regulatory effect of resveratrol on autophagy in osteoblasts and osteoclasts. The inhibition of autophagy in the osteoblasts and its activation in the osteoclasts, as shown in postmenopausal osteoporosis, was reversed with the resveratrol treatment. This mechanism provided new insight into the use of resveratrolin postmenopausal osteoporosis.

### Reagents

HNO_3_, trypsin, collagenase type I, resveratrol, bafilomycin, leupeptin, PMSF and DAPI were obtained from Sigma Chemical Company (CA, USA). α-MEM solution and DMEM medium were purchased from Gibco (NY, USA). Paraformaldehyde and RIPA lysis buffer were gained from Solarbio (Beijing, China). BCA assay kit was purchased from Thermo Fisher Scientific (MA, USA).

## Methods

### Resveratrol intervention and grouping

A total of 50 female Sprague Dawley (SD) rats weighing 250 ± 10 g, 10–12 weeks old, were purchased from the laboratory animal center (Nanjing, China). One group was sham-operated (*n* = 10), and the second group was ovariectomized (*n* = 40) [23]. One week after surgery, the OVX group was randomly divided into 4 groups as follows: 1) OVX group; 2) OVX group+ 10 mg/kg/d resveratrol; 3) OVX group+ 20 mg/kg/d resveratrol; and 4) OVX group+ 40 mg/kg/d resveratrol. Resveratrol was dissolved in 5 ml of normal saline and administered to rats intragastrically for 8 weeks.

### Determination of the calcium content in the right femur of the rats

The right femur of each rat was dissected, and the attached tissue was removed. The femur was then dehydrated and dried for approximately 72 h. All the samples had approximately the same weight and were all kept in a constant temperature drying oven at (80 °C). Afterwards, the net weight of the dry bone tissue was measured. The bone tissue was then dissolved with 2 mL HNO_3_, and the Ca^2+^ concentration was determined by atomic absorption spectrophotometry [24] after a 400-fold dilution. Subsequently, this was converted to bone calcium content using the following formula: bone calcium content = Ca^2+^ concentration (μg/mL) × 400.

### Determination of the bone mineral density of rats

The dual-emission X-ray absorptiometry (DEXA, Hologic, USA) was used to determine the BMD by scanning the lumbar vertebrae (L3) and the right distal femur-tibia bone region of the rats [25]. All the samples were scanned in the same region orientation. The BMD was measured by the software in the region of interest on 3 different occasions, and the calculated average value was used (g/cm^2^).

### Isolation of osteoblasts from the rats

The osteoblasts were isolated from the rat lumbar vertebrae (L1-L5) by enzyme digestion. After the rats were sacrificed, the lumbar vertebrae (L1-L5) were obtained under aseptic conditions and were cut into 1 mm^3^ small pieces, digested with 0.25% trypsin (Sigma, USA) for 20 min (37 °C), and further digested with 0.2% collagenase type I (Sigma, USA) and 0.25% trypsin for 20 min, with a total of 5 cycles of digestion. The 3rd to 5th digested cell suspension were collected and centrifuged at 1000 r/min for 10 min, and the cell sedimentation was the osteoblasts.

### Isolation of osteoclasts from the rats

The osteoclasts were isolated from the rat lumbar vertebrae (L1-L5) in each group by bone marrow induction. After the rats were sacrificed, the lumbar vertebrae (L1-L5) were obtained under aseptic conditions. The vertebral column was cut longitudinally, the bone marrow cavity surface was then washed by an α-MEM solution (Gibco, USA), and the washing liquid was collected by cell sieve filtration (Corning, USA). Afterwards, the vertebrae were quickly cut into pieces, and transferred to the centrifuge tube, together with the collected rinsing liquid. These were shaken for 5 s and were left to stand for 7 s. The upper suspension was collected by filtration through a cell sieve into a new centrifuge tube. The above mentioned steps were repeated 3 times to collect the cell suspension. The suspension was centrifuged at 1000 r/min for 10 min, and the cell sedimentation was the osteoclasts.

### Primary cell culture and intervention

The osteoblasts and osteoclasts isolated from the rats were cultured in DMEM medium (Gibco, USA) and were stimulated with 10 μM, 20 μM, and 50 μM resveratrol (Sigma, USA) for 96 h. After the differentiation, 400 nM bafilomycin (Sigma, USA) or 40 μM leupeptin (Sigma, USA) was administered for 4 h to inhibit autophagy. The osteoblasts were divided into 8 groups as follows: 1) sham group; 2) OVX group; 3) OVX group+ 10 μM Resveratrol; 4) OVX group+ 20 μM Resveratrol; 5) OVX group+ 50 μM Resveratrol; 6) OVX group+ 10 μM Resveratrol+ Bafilomycin; 7) OVX group+ 20 μM Resveratrol+ Bafilomycin; and 8) OVX group+ 50 μM Resveratrol+ Bafilomycin. The osteoclasts were also divided into 8 groups as follows: 1) sham group; 2) OVX group; 3) OVX group+ 10 μM Resveratrol; 4) OVX group+ 20 μM Resveratrol; 5) OVX group+ 50 μM Resveratrol; 6) OVX group+ 10 μM Resveratrol+ Leupeptin; 7) OVX group+ 20 μM Resveratrol+ Leupeptin; and 8) OVX group+ 50 μM Resveratrol+ Leupeptin.

### Western blotting

A total of 200 μL of RIPA lysis buffer (Solarbio, Beijing, China) and 2 μL of PMSF (Sigma, USA) were added to the culture dishes, and the cells were scraped gently from the dishes. The cell suspension was then ultrasonicated on ice. After centrifugation, the supernatant was collected, and the protein concentrations were measured using a BCA assay kit (Thermo, USA). The 5x loading buffer was added in a volume ratio of 1:5, and the samples boiled for 10 min. Later on, the processed protein samples were sequentially added to the wells and were electrophoresed at 80 V for 2 h. The transfer was kept at a 200 mA steady flow for 1.5 h. Antibodies against VEGF (Proteintech, USA, 19003–1-AP), TNFSF11 (Abcam, USA, ab100749), COL1A1 (Proteintech, USA, 14695–1-AP), BGLAP (Abcam, USA, IG272465), GAPDH (Santa Cruz, USA, ab4531), RANKL (Abcam, USA, ab9957), p62 (Proteintech, USA, 55274–1-AP), LC3I/II (Abcam, USA, XY-ABD101), atg5 (Proteintech, USA, 66744–1-Ig), atg7 (Proteintech, USA, 11262–2-AP), and atg12 (Proteintech, USA, 11122–1-AP) were used for the Western blotting. GAPDH was used as an internal control protein. Band density was quantified using Image J software. The relative levels of proteins were normalized to GAPDH.

### Immunofluorescence staining

The cells were fixed with 4% paraformaldehyde (Solarbio, Beijing, China) for 15 min at room temperature, washed with 1 × PBS, and permeabilized and blocked (Solarbio, Beijing, China) at 37 C incubator for 10 min, and then incubated with the LC3 antibody (Abcam, USA) at 4 C overnight. A fluorescent secondary antibody at a dilution of 1:200 was used for incubation at 37 C for 1 h. Finally, the cells were incubated with DAPI (1:1000) (Sigma, CA, USA) for 10 min after 1 × PBS washing and were then covered with glycerin.

### Statistical analysis

GraphPad Prism software 6.0 (CA, USA) was used for the data analyses. The data are presented as the mean ± SD. A one-way ANOVA was used for multiple comparisons followed by Bonferoni test. *P* < 0.05 was considered statistically different.

## Results

### Effect of resveratrol on the bone calcium content and bone mineral density in OVX rats

**Fig. 1:**
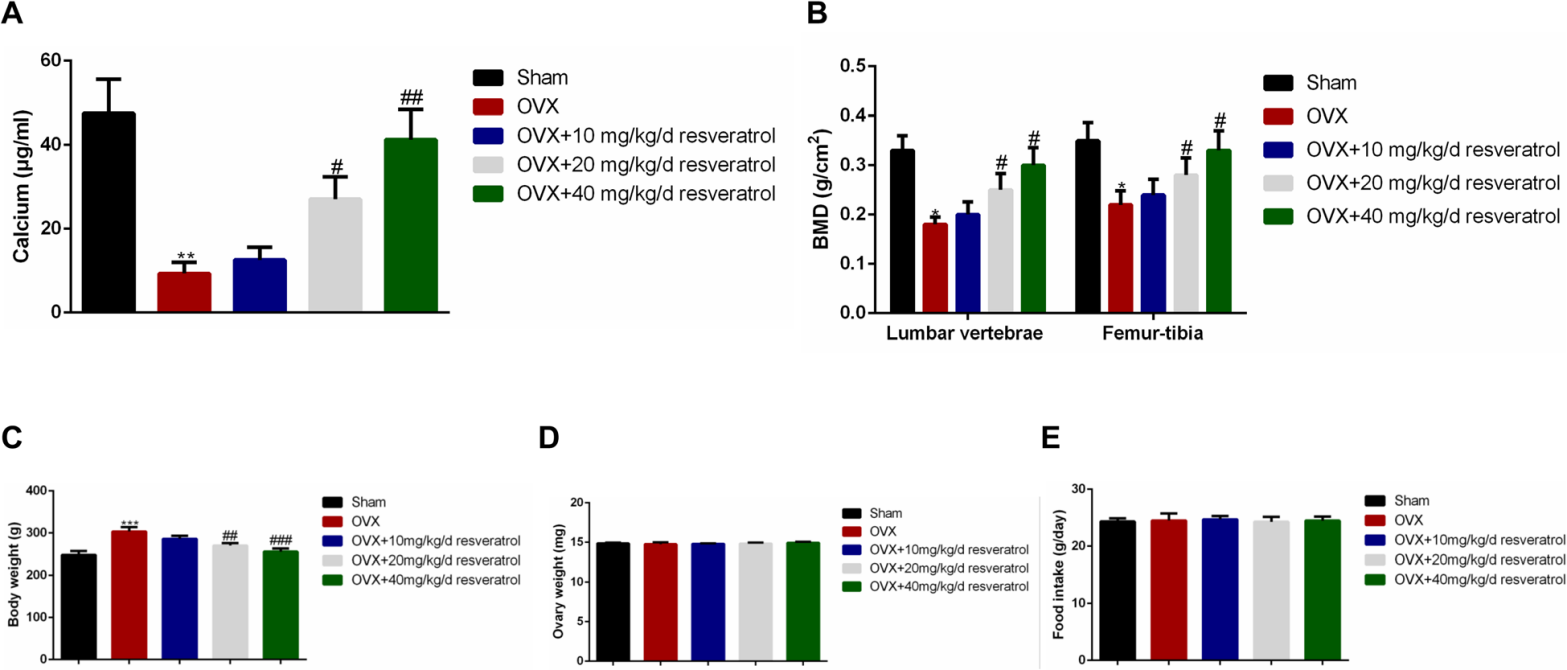
Effect of resveratrol on femur calcium content and bone mineral density in OVX rats. An ovariectomy (OVX) was performed in rats, and then the rats were given different doses of resveratrol. **a** The right femurs of the rats were used to determine the bone calcium content; **b** The lumbar vertebrae (L3) and right distal femur-tibia of rat were scanned by dual-emission X-ray absorptiometry, and the bone mineral density (BMD) was measured by three independent experiments. **c** The body weight of rats in the five groups was determined after resveratrol treatment for 8 weeks. **d** The ovary weights of rats in the five groups were shown. **e** The food intake of rats in the five groups was shown after resveratrol treatment for 8 weeks. *n* = 8 for each group. The data were shown as the mean ± SD (*n* = 8). Compared to the Sham group, *p < 0.05, **p < 0.01, ***p < 0.001; Compared to the OVX group, *# p < 0.05, ## p < 0.01, ### p < 0.001*

To determine whether resveratrol restored bone loss caused by estrogen deficiency, the OVX rats were treated with different doses of resveratrol for 8 weeks. As shown in Fig. 1a, compared to the Sham group, the bone calcium content in the right femur was significantly decreased in the OVX group (*p* < 0.05). However, administering 10 mg/kg/d (low-dose), 20 mg/kg/d (medium-dose), and 40 mg/kg/d (high-dose) resveratrol to the OVX rats did dramatically alter the phenomenon with the bone calcium content measured at 12.56 ± 3.01 and 27.06 ± 5.36, 41.27 ± 7.13 μg/mL, respectively. Moreover, the bone calcium content of the medium and high dosage group (20 mg/kg/d and 40 mg/kg/d) was much higher than the OVX group (*p* < 0.05). For the BMD measurement by DEXA, figures showed that the BMD was markedly reduced in both the lumbar vertebrae and femur-tibia of the OVX group compared to the Sham group (Fig. 1b). Even so, resveratrol was able to reverse the decrease in BMD of the OVX rats in a dose-dependent manner. The BMD of OVX rats given the medium and high dosage (20 mg/kg/d and 40 mg/kg/d) were apparently higher than the OVX group (*p* < 0.05). Besides, the body weight of rats in OVX group was significantly increased compared to that in the sham group (*p* < 0.05), which was reversed by 20 mg/kg/d (*p* < 0.01) and 40 mg/kg/d of resveratrol (*p* < 0.001) respectively (Fig. 1c). The weight of the ovaries excised from the rats in the five groups showed no significant differences (Fig. 1d). Furthermore, results revealed that there were no significant differences in food intake among the five groups (Fig.1e). These data indicate that the OVX rats presented with noticeable bone loss and weight gain. Overall, treatment with resveratrol had a beneficial effect on postmenopausal osteoporosis.

### Effect of resveratrol on the differentiation of osteoblasts and osteoclasts

**Fig. 2:**
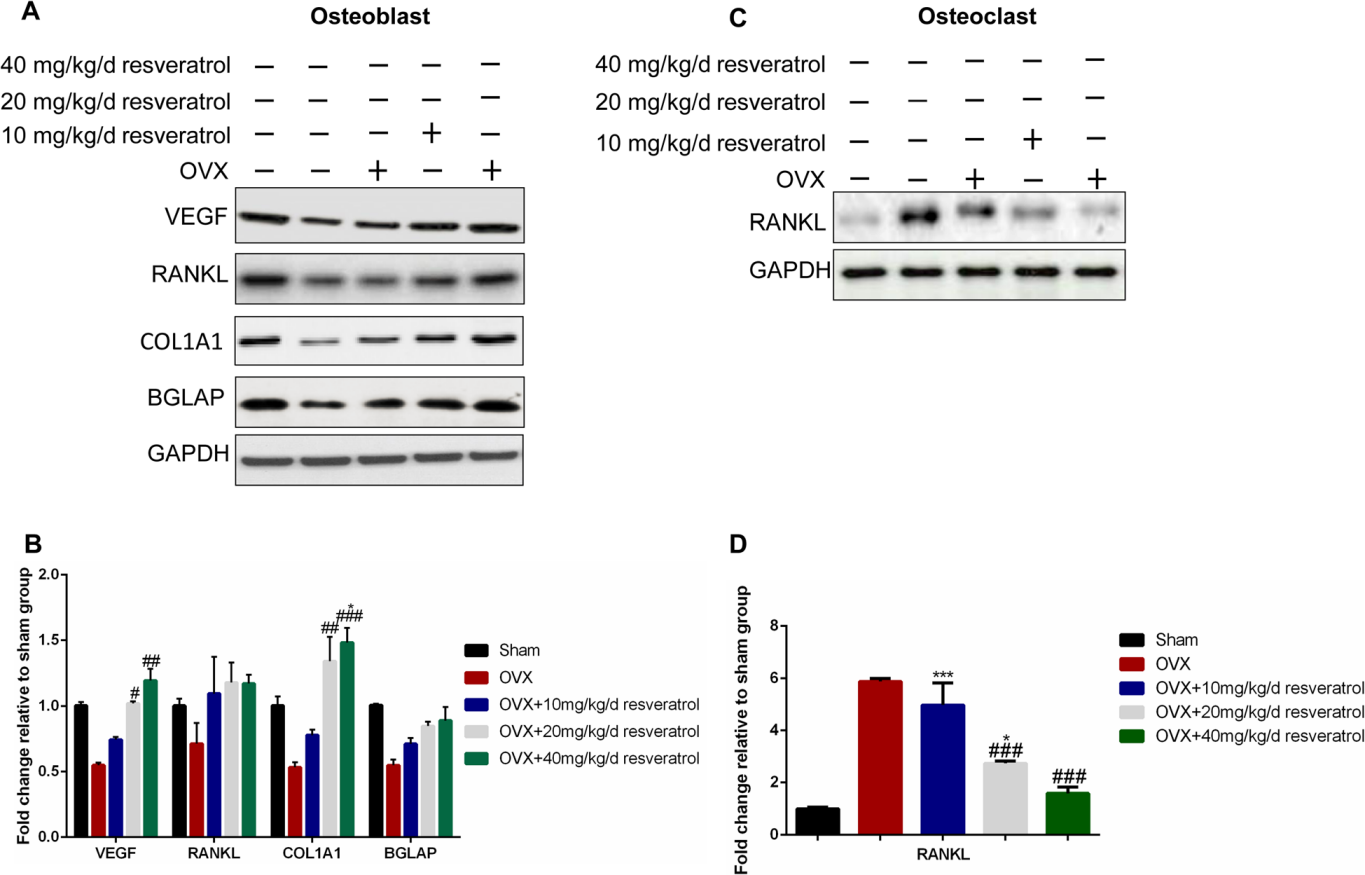
Effect of resveratrol on the differentiation of osteoblasts and osteoclasts. **a** and **b** The osteoblasts were isolated from the rat lumbar vertebrae (L1-L5) in each group, and the protein levels of VEGF, TNFSF11, COL1A1, and BGLAP were measured by Western blotting; **c** and **d** The osteoclasts were isolated from the rat lumbar vertebrae (L1-L5) in each group, and the protein level of RANKL was measured by Western blotting. VEGF, vascular endothelial growth factor; TNFSF11, tumor necrosis factor ligand superfamily member 11; COL1A1, alpha-1 type I collagen; BGLAP, bone glaprotein; RANKL, receptor activator of nuclear factor-κB ligand. The data were shown as the mean ± SD (*n* = 3). Compared to the Sham group, *p < 0.05, **p < 0.01, ***p < 0.001; Compared to the OVX group, *# p < 0.05, ## p < 0.01, ### p < 0.001*

To investigate the effect of resveratrol on the differentiation of osteoblasts and osteoclasts, cells were isolated. As exhibited in Fig. 2, the osteoblast differentiation-related proteins, including vascular endothelial growth factor (VEGF) and alpha-1 type I collagen (COL1A1), were markedly decreased, while the osteoclast differentiation related protein receptor activator of nuclear factor-κB ligand (RANKL) was significantly increased (*p* < 0.05). The addition of resveratrol as an intervention up-regulated the protein expressions of VEGF & COL1A1 and down-regulated the protein level of RANKL in a dose-dependent manner. These results demonstrated that the estrogen deficiency in the OVX rats led to inactive bone formation, active bone destruction, and dissolution, while resveratrol reversed the imbalance between bone formation and destruction.

### Effect of resveratrol on the regulation of autophagy in osteoblasts and osteoclasts

**Fig. 3:**
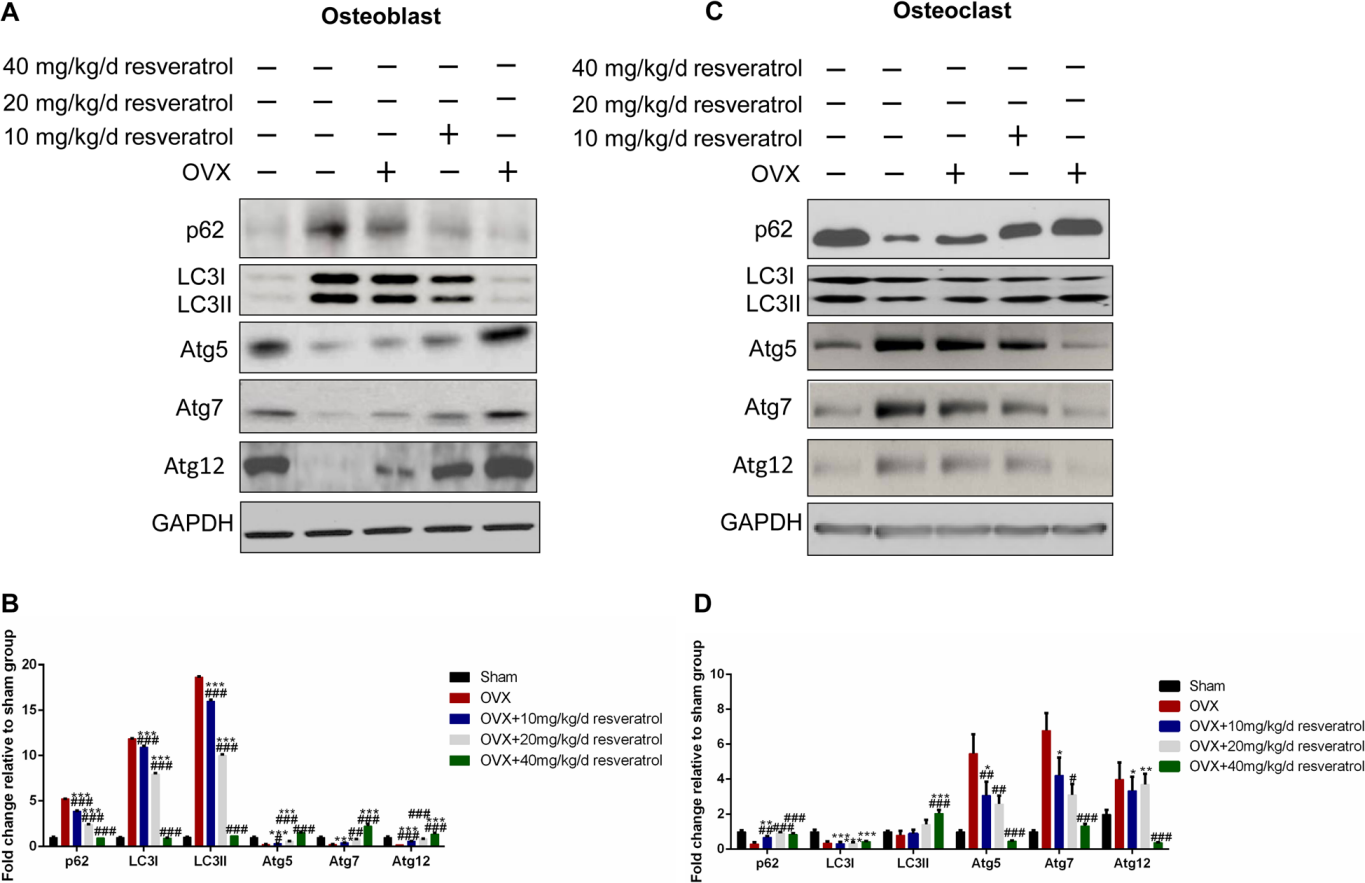
Effect of resveratrol on the regulation of autophagy in osteoblasts and osteoclasts. **a** and **b** The osteoblasts were isolated from the rat lumbar vertebrae (L1-L5) in each group, and the protein levels of p62, LC3I/LC3II, atg5, atg7, and atg12 were measured by Western blotting; **c** and **d** The osteoclasts were isolated from the rat lumbar vertebrae (L1-L5) in each group, and the protein levels of p62, LC3I/LC3II, atg5, atg7, and atg12 were measured by Western blotting. LC3, protein microtubule-associated protein 1 light chain-3; Atg, autophagy related gene. The data were shown as the mean ± SD (*n* = 3). Compared to the Sham group, *p < 0.05, **p < 0.01, ***p < 0.001; Compared to the OVX group, *# p < 0.05, ## p < 0.01, ### p < 0.001*

To further investigate the mechanism of the regulatory effect of resveratrol on osteoblast and osteoclast differentiation, we tested autophagy-related protein levels in each group. As shown in Fig. 3a and b, compared to the sham group, the protein levels of atg5, atg7, and atg12 in the osteoblasts from the OVX group were significantly decreased, and the expression of p62 increased. This indicated that autophagy was inhibited and the autophagy flux was reduced. An increase in LC3II protein refers to the decreased degradation of LC3II. The resveratrol activated autophagy in a dose-dependent manner, which was shown by the increased levels of atg5, atg7, and atg12 and the decreased level of p62. Conversely, compared to the sham group, the protein levels of atg5, atg7, and atg12 in the osteoclasts from the OVX group were significantly increased, and the expression of p62 was decreased, indicating that autophagy was activated and autophagy flux increased. The resveratrol intervention inhibited autophagy in a dose-dependent manner (Fig. 3c and d). These results demonstrated that having an estrogen deficiency inhibited osteoblast differentiation by suppressing autophagy and promoting osteoclast differentiation by activating autophagy. In addition, resveratrol restored the balance of bone formation and bone resorption by regulating autophagy in both osteoblasts and osteoclasts.

### Autophagy modulation in cultured osteoblasts and osteoclasts with resveratrol stimulation

**Fig. 4:**
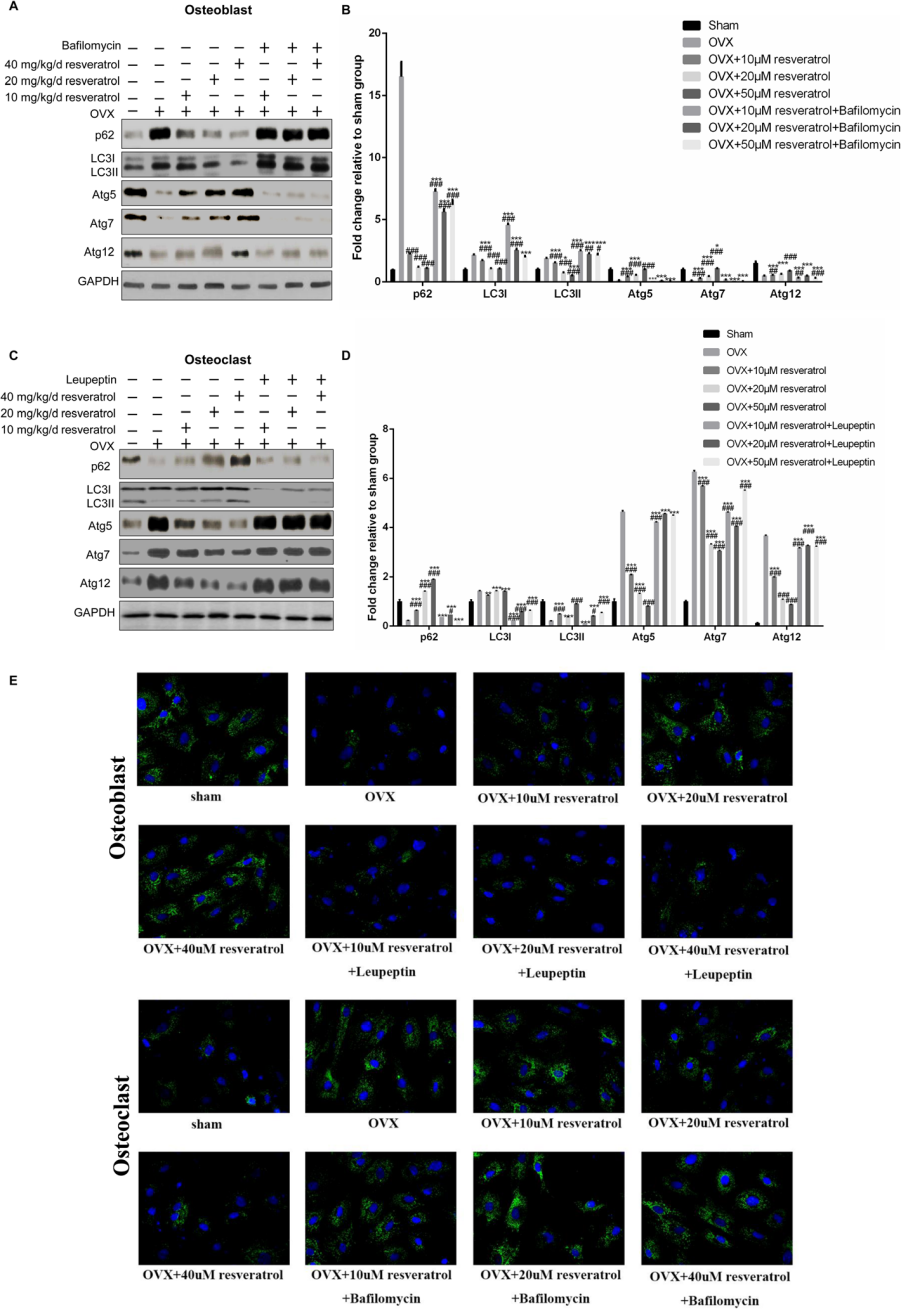
The autophagy modulation in cultured osteoblasts and osteoclasts with resveratrol stimulation. The cells were cultured in a differentiation medium and were stimulated with resveratrol. After differentiation, autophagy was inhibited or activated. The protein levels of p62, LC3I/LC3II, atg5, atg7, and atg12 were then measured by Western blotting in the (**a**, **b**) osteoblasts and (**c**, **d**) osteoclasts; **e** The fluorescence intensity of LC3 was measured in each group. LC3, protein microtubule-associated protein 1 light chain-3; atg, autophagy related gene. The data were shown as the mean ± SD (*n* = 3). Compared to the Sham group, *p < 0.05, **p < 0.01, ***p < 0.001; Compared to the OVX group, *# p < 0.05, ## p < 0.01, ### p < 0.001*

To further uncover the mechanism of resveratrol by in vitro experiments, the cells were cultured in a differentiation medium and were stimulated with 10 μM, 20 μM, and 50 μM resveratrol for 96 h. After differentiation, 400 nM bafilomycin or 40 μM leupeptin was added for 4 h to inhibit autophagy. We found that resveratrol activated and inhibited autophagy in a dose-dependent manner in the cultured osteoblasts and osteoclasts, as assessed by the expression of autophagy-related proteins (Fig. 4a-d) and the fluorescence intensity of LC3 (Fig. 4e). With the addition of bafilomycin, the increased autophagy in the osteoblasts disappeared. Conversely, with the addition of leupeptin, the inhibitory effect of autophagy in the osteoclasts was reversed (Fig. 4a-d). These results again demonstrated that the effect of resveratrol on osteoblast and osteoclast differentiation was achieved by the regulation of autophagy levels.

## Discussion

Postmenopausal osteoporosis is a metabolic disease in which bone resorption by osteoclasts is more active than bone formation from osteoblasts due to estrogen deficiency [1]. Resveratrol has been used for estrogen replacement therapy in postmenopausal osteoporosis. However, the underlying mechanism of resveratrol for postmenopausal osteoporosis treatment is not well known. Therefore, the present study was aimed to explore the molecular mechanism of resveratrol on osteoblastic differentiation in rats.

The ovariectomized rat model was first established to induce osteoporosis in this study, which was approved by the Food and Drug Administration (FDA) [26, 27]. In the ovariectomized rat, the calcium content and BMD was decreased in the study. These results were consistent with previous studies [28, 29]. Tsai et al. revealed that the calcium mechanism was altered in OVX rats [28]. Comelekoglu et al. found that BMD was reduced in 14% of OVX rats compared to control rats [29]. Moreover, accumulated evidences proved that osteoporosis was usually accompanied by weight gain [30, 31]. Similar to these reports, we found that the body weight of OVX rats was elevated without altering food intake. Hence, the ovariectomized rat model was successfully established. The OVX rats presented bone loss and weight gain. In addition, the resveratrol treatment alleviated osteoporosis symptoms, which were in agreement with other publications [32, 33].

To investigate the regulation mechanism of resveratrol on postmenopausal osteoporosis, the effects of resveratrol in the differentiation of osteoblasts and osteoclasts was determined. The expression levels of VEGF, RANKL, COL1A1, and BGLAP were decreased in OVX rats, while the effects were abolished by resveratrol. Hu et al. found that osteoblast-derived VEGF promoted osteoblast differentiation at bone-repair sites [34]. RANKL was considered as the osteoclast development when binding to RANK [35], while RANKL also promoted osteoblast differentiation and osteoblastogenesis [36]. COL1A1 was the mark of osteoclasts, and the expression of COL1A1 was increased during osteoblast differentiation [37]. BGLAP was also an osteogenic marker, and participated in the intermediate or late stages of osteoblast differentiation [38]. In osteoblasts from OVX rats, the level of RANKL was increased, which was reversed by resveratrol. Thus, based on the evidence above, we concluded that resveratrol promoted osteoblastic differentiation and suppressed osteoclastic differentiation in postmenopausal osteoporosis rats. The results were consistent with the reported regulatory effects of resveratrol on osteoblasts and osteoclasts [39, 40]. Ma et al. found that resveratrol rescued the suppression of osteoblast differentiation caused by LPS [39]. In addition, PCL and poly (lactic) acid (PLA) loading resveratrol inhibited osteoclast differentiation [40].

Previous studies proved that some biological process regulated osteoblast differentiation including autophagy [41]. In this study, autophagy was activated during the osteoblast differentiation process [42]. In addition, oxidative stress increased and autophagy counterbalanced this during aging, making autophagy an important factor in postmenopausal osteoporosis [43]. We speculated that autophagy might be involved in the regulation of resveratrol on differentiation of osteoblasts. Hence, we detected the changes of autophagy-related proteins including p62, LC3, Atg5, Atg7 and Atg 12 and autophagy flux in the osteoblasts. Results showed that the protein levels of Atg5, Atg7 and Atg 12 and autophagy flux were decreased, while the protein level of p62 was increased in the OVX osteoblasts. With the resveratrol intervention, the autophagy-related protein changes were reversed. These changes indicated that the resveratrol activated autophagy had increased the autophagy flux in osteoblasts. Interestingly, the expression of LC3II in the OVX osteoblasts was significantly up-regulated when autophagy was inhibited, thus this expression was up-regulated after being treated with resveratrol. Traditionally, LC3II is a classical marker of a mature autophagosome, and its expression is positively correlated with the autophagy level [44]. Recently, it was shown that an intracytoplasmic increase of LC3II was caused by an autophagy inhibition, which led to the suppression of LC3II degradation [45].

Even so, several studies show that autophagy-related proteins are involved in the differentiation and formation of osteoclasts [46]. Osteoclast-mediated bone resorption is markedly suppressed by the autophagy inhibitor Bafilomycin [47]. Hence, we speculated that autophagy might be involved in the regulation of resveratrol on osteoclast differentiation. We detected the changes of autophagy-related proteins including p62, LC3, Atg5, Atg7, Atg 12, and autophagy flux in the osteoclasts. Our investigation revealed that the protein levels of Atg5, Atg7, and Atg12 and autophagy flux were markedly decreased, while the protein levels of p62 and LC3II were increased in the OVX osteoclasts. With resveratrol intervention, the autophagy-related protein changes were reversed, indicating that resveratrol inhibited autophagy and decreased the autophagy flux in osteoclasts. The regulatory effects of resveratrol on autophagy have been reported in various diseases. For example, in neurodegenerative disorders, resveratrol activates cellular autophagy to prevent apoptosis under inflammation and oxidative stress [48]. Also, adipose stem cells (ASCs) show a recovered stemness and multipotency after resveratrol treatment in metabolic syndrome (MS), which occurs via autophagic regulation [49]. Moreover, resveratrol activates the SIRT-1 pathway to attenuate ER stress, preventing HepG2 cell apoptosis [50]. This is consistent with our results, indicating that resveratrol regulates autophagy. However, how resveratrol activates or inhibits autophagy in a cell dependent manner, namely the osteoblast vs osteoclast, remains to be determined.

Previously, details of the mechanisms of action of resveratrol on bone tissue were limited. Literature suggests that it affects osteoclasts and osteoblasts either directly or indirectly by stimulating bone formation and decreasing bone resorption [51]. Our findings substantiate this notion and provide further information on the role of resveratrol for the treatment or prevention of the damage that occurs due to postmenopausal osteoporosis. These findings also support further studies to examine the in vivo function of resveratrol and its effect on bone tissue, which may support the development of studies for clinical trials involving postmenopausal osteoporosis.

## Conclusion

In general, we demonstrated that resveratrol had beneficial effects on postmenopausal osteoporosis. It mainly functioned by promoting osteoblast differentiation and suppressing osteoclast differentiation, which led to more bone formation and less bone resorption. Importantly, the underlying mechanism of resveratrol was through the activation of autophagy in osteoblasts and the inhibition of autophagy in osteoclasts. These findings may provide a new strategy for postmenopausal osteoporosis therapy.

## Data Availability

All the data generated or analyzed during this study are included in this published article.

## Abbreviations

OVX: Ovariectomy
SD: Sprague Dawley
BMD: Bone mineral density
VEGF: Vascular endothelial growth factor;
TNFSF11: Tumor necrosis factor ligand superfamily member 11
BGLAP: Bone glaprotein
RANKL: Receptor activator of nuclear factor-κB ligand
